# Computational prediction and experimental validation associating FABP-1 and pancreatic adenocarcinoma with diabetes

**DOI:** 10.1186/1471-230X-11-5

**Published:** 2011-01-20

**Authors:** Ravi N Sharaf, Atul J Butte, Kelli D Montgomery, Reetesh Pai, Joel T Dudley, Pankaj J Pasricha

**Affiliations:** 1 Department of Gastroenterology and Hepatology, Stanford University School of Medicine, Alway Building, Room M211, 300 Pasteur Drive, MC: 5187, Stanford, CA, 94305-5187, USA; 2 Department of Pediatrics, Stanford University School of Medicine, 300 Pasteur Drive, Room H310, Stanford, CA, 94305-5208, USA; 3 Lucile Packard Children's Hospital, 725 Welch Road, Palo Alto, CA, 94304, USA; 4 Biomedical Informatics Graduate Training Program, Stanford University School of Medicine, 251 Campus Drive, MSOB, x-215, MC: 5479, Stanford, CA, 94305-5479, USA; 5 Department of Pathology, Stanford University School of Medicine, 300 Pasteur Drive, Lane 235, Stanford, CA, 94305-5324, USA

## Abstract

**Background**

Pancreatic cancer, composed principally of pancreatic adenocarcinoma (PaC), is the fourth leading cause of cancer death in the United States. PaC-associated diabetes may be a marker of early disease. We sought to identify molecules associated with PaC and PaC with diabetes (PaC-DM) using a novel translational bioinformatics approach. We identified fatty acid binding protein-1 (FABP-1) as one of several candidates. The primary aim of this pilot study was to experimentally validate the predicted association between FABP-1 with PaC and PaC with diabetes.

**Methods**

We searched public microarray measurements for genes that were specifically highly expressed in PaC. We then filtered for proteins with known involvement in diabetes. Validation of FABP-1 was performed via antibody immunohistochemistry on formalin-fixed paraffin embedded pancreatic tissue microarrays (FFPE TMA). FFPE TMA were constructed using148 cores of pancreatic tissue from 134 patients collected between 1995 and 2002 from patients who underwent pancreatic surgery. Primary analysis was performed on 21 normal and 60 pancreatic adenocarcinoma samples, stratified for diabetes. Clinical data on samples was obtained via retrospective chart review. Serial sections were cut per standard protocol. Antibody staining was graded by an experienced pathologist on a scale of 0-3. Bivariate and multivariate analyses were conducted to assess FABP-1 staining and clinical characteristics.

**Results**

Normal samples were significantly more likely to come from younger patients. PaC samples were significantly more likely to stain for FABP-1, when FABP-1 staining was considered a binary variable. Compared to normals, there was significantly increased staining in diabetic PaC samples (p = 0.004) and there was a trend towards increased staining in the non-diabetic PaC group (p = 0.07). In logistic regression modeling, FABP-1 staining was significantly associated with diagnosis of PaC (OR 8.6 95% CI 1.1-68, p = 0.04), though age was a confounder.

**Conclusions**

Compared to normal controls, there was a significant positive association between FABP-1 staining and PaC on FFPE-TMA, strengthened by the presence of diabetes. Further studies with closely phenotyped patient samples are required to understand the true relationship between FABP-1, PaC and PaC-associated diabetes. A translational bioinformatics approach has potential to identify novel disease associations and potential biomarkers in gastroenterology.

## Background

Pancreatic cancer, composed principally of pancreatic adenocarcinoma (PaC), is the fourth leading cause of cancer death in the United States. At diagnosis, more than 85% of patients with PaC have unresectable disease, with a median survival of 4-6 months [1,2].

PaC is a diabetogenic state with 45-65% of PaC patients having diabetes mellitus (DM). New-onset DM, occurring within approximately 2 years prior to cancer diagnosis, may be a paraneoplastic effect of the tumor itself secondary to a circulating tumor-associated protein that causes insulin resistance and beta-cell dysfunction [3]. Adult patients with new onset DM have an 8-fold increased risk of developing PaC [4]. Given that this PaC-associated DM often occurs when the cancer is asymptomatic and resectable, it may be a useful marker of early disease, though it is difficult to clinically distinguish from chronic type II diabetes mellitus [3].

Several strategies have been used to identify pancreatic cancer-associated diabetogenic factors, including microarray work, genotyping, quantitative real time polymerase chain reaction, immunohistochemistry, serum analysis, gel electrophoresis and cell culture, and serum radioimmunoassay. These have yielded putative biomarkers, including connexin-26, insulin gene promoter polymorphisms, glucagon/insulin ratio, S1000-A8 calcium binding protein, islet amyloid polypeptide, and insulin-like growth factor-I [5-10]. None have proven definitive. The challenge is in identifying a molecule that is specifically upregulated in pancreatic adenocarcinoma that simultaneously leads to diabetes.

We sought to identify molecules associated with PaC and PaC with diabetes (PaC-DM) using an integrative genomics approach, building from our previous methods in intersecting publicly-available gene expression measurements to find DNA variants associated with disease [11,12]. We identified fatty acid binding protein-1 (FABP-1) as one of several candidate molecules. The primary aim of this pilot study was to experimentally validate the predicted association between FABP-1 and PaC and PaC with diabetes.

## Methods

### Integrative Genomics

We have previously described the creation of a database of gene expression microarray experiments across human diseases, built from publicly-available repositories [13]. Specifically, gene expression microarray experiments in the NCBI Gene Expression Omnibus (GEO) characterizing human disease conditions were automatically identified using a method where the Medical Subject Heading (MeSH) terms attributed to publications associated with GEO experiments are were evaluated for disease concepts using the Unified Medical Language System (release 2007AC) [14,15]. Each of these experimental data sets determined to be relevant to a human disease based on associated MeSH disease concepts was subject to automated annotation of the disease condition, the tissue or biological substance from which the samples were derived, and whether or not the experiment measured a normal control state complimentary to the annotated disease state. The automated annotation step was performed using a previously published method that analyzes particular annotations in a GEO DataSet, which is a higher-order representation of an experiment in GEO that groups experimental samples into logical subsets (e.g. "control" and "treatment") using a free-text vocabulary [16]. The disease and tissue annotations were manually reviewed in a post-processing step to ensure accuracy.

Our resulting data set incorporates over 200 diseases studied across over 350 GEO datasets comprising nearly ten thousand individual microarrays, studied in over 100 tissues. For every gene in the genome measured across microarray platforms, we calculated a change in rank normalized expression level in that gene from normal samples to disease samples, resulting in a calculated measure ranging from +1.0 (a gene changing from lowest measured expression level in normal samples to highest measured expression level in disease samples) to -1.0 (a gene changing from highest measured expression level in normal samples to lowest measured expression level in disease samples).

Within this database of disease-related gene expression changes, we searched for genes selectively upregulated in pancreatic tissue from pancreatic adenocarcinoma, but not in pancreatic tissue from samples with acute pancreatitis and type I diabetes mellitus; comparison groups were limited to those conditions with robust available GEO data.

Of those genes selectively fitting this profile, the rank normalized expression level for the top ten genes ranged from 0.19-0.12 with FABP-1 measuring 0.14, fifth on the list.

We then compared this list of genes selectively upregulated in pancreatic tissue from pancreatic adenocarcinoma with that of genes known to be associated with type II DM from the NIH Genetic Association Database http://geneticassociationdb.nih.gov/. Type II DM susceptibility genes were compared given the similar phenotype in pancreatic cancer-associated diabetes of beta-cell dysfunction and insulin resistance [17]. Type II diabetes susceptibility genes were derived using the same bioinformatics methods described above.

With a list of genes upregulated in both pancreatic adenocarcinoma and type II DM, we then filtered for a biofluid database containing proteins known to be detectable in the blood or urine, integrating protein lists from the Plasma Proteome Institute, the MAPU Proteome database, and the Urinary Exosome database [11,18-20]. We narrowed our search to proteins present in serum/urine because of the likely necessity for a protein synthesized in the tumor to use the blood to reach insulin target tissues, such as fat, muscle, and liver. FABP-1 was thus chosen as a candidate for validation.

### Selection of Cases and TMA

Formalin fixed paraffin embedded (FFPE) Tissue Microarrays (TMA) were constructed using 148 cores of pancreatic tissue from 134 patients collected by the Stanford University Department of Pathology between 1995 and 2002 from patients who underwent pancreatic surgery. The FFPE TMA were constructed in 2004 using a manual tissue arrayer (Beecher Instruments, Silver Spring, Maryland, United States) following previously described techniques using 1.0 mm cores [21]. The cores were taken from areas in the paraffin block that were representative of the diagnostic tissue.

**Figure 1:**
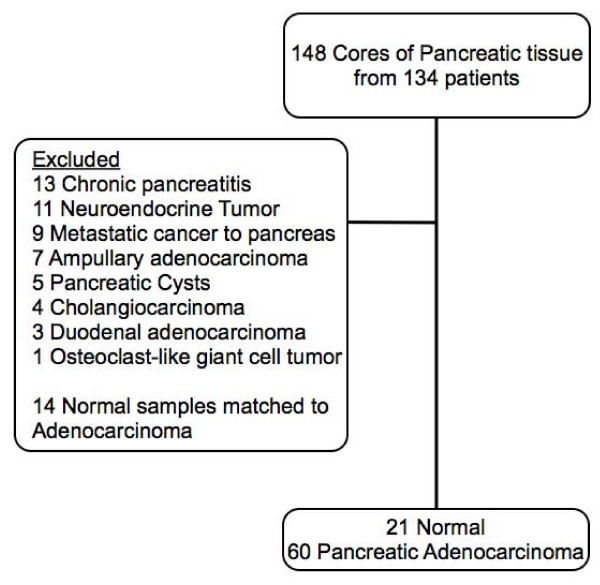
**Study flowchart**.

Figure 1 shows a flow diagram of samples included and excluded from the analysis. Excluded were 67 samples, consisting of chronic pancreatitis (13), neuroendocrine tumors (11), metastatic cancer to pancreas (9), ampullary adenocarcinoma (7), pancreatic cysts (5), cholangiocarcinoma (4), duodenal adenocarcinoma (3), and osteoclast-like giant cell tumor (1).

Biopsies of normal pancreatic tissue are not routinely obtained. Normal samples on our TMA were derived from histologically normal cores obtained from an organ that often also contained a pathologic pancreatic condition. This is common practice in construction of tissue microarrays [21-23]. In this way, many normal tissues were "matched" to a given pancreatic disease state. The TMA contained 14 normal samples matched to pancreatic adenocarcinoma. In a matched analysis, it is the discordant or differentially-staining pairs that are informative for analysis. However, in our 14 normal/pancreatic adenocarcinoma matched samples, only 4 were discordant for staining. We thus had inadequate power to conduct a matched statistical analysis. We consequently decided to compare FABP-1 staining amongst "non-matched" samples: PaC samples and normal samples derived from an organ that did not also contain pancreatic adenocarcinoma. Of the 21 normal samples we used in our analysis, 5 were not matched to any other pancreatic pathology. The other normal samples were matched to non PaC conditions: 8 normal/neuroendocrine tumors, 2 normal/ampullary cancers, 1 normal/metastatic cancer to the pancreas, 4 normal/pancreatic cysts, and 1 normal/cholangiocarcinoma. Primary analysis was performed on 21 normal and 60 pancreatic adenocarcinoma samples, stratified for diabetes.

IRB approval was obtained from the Stanford Research Compliance Office, allowing retrospective collection of patients' age, gender, presence of diabetes, alcohol and tobacco use. Clinical data was collected on a standardized form by one physician-abstractor (RNS). Clinical data was obtained from notes in a given patient's chart.

### Immunohistochemistry Staining and Scoring

Serial sections of 4 um were cut from the TMA blocks, deparaffinized in xylene, and hydrated in a graded series of alcohol. The slides were pretreated with EDTA pH9 buffers for FABP-1 in a microwave step. Immunohistochemistry (IHC) staining was then performed using the DAKO EnVision + System, Peroxidase (DAB), (DAKO, Cambridgeshire, United Kingdom) for FABP-1 (1:20 dilution, Abcam, Cambridge, MA). Antibody staining was graded by a single experienced pathologist per standard protocol on a scale of 0-3 (0 = no staining, 1+ = 1-25%, 2+ = 25-75%, 3+ = > 75%) based on the extent of cytoplasmic staining in an individual histologic core [22]. FABP-1 staining was interpreted as a binary variable (0 or ≥ 1) as well as a FABP-1score (number between 0-3).

### Statistical Analyses

Patient data was reported using bivariate (χ^2^, Fishers Exact test, Wilcoxon Rank Sum with Bonferroni correction, and non parametric Kruskal-Wallis ANOVA) and multivariate analyses (logistic regression with binary outcome variables) to assess FABP-1 staining and clinical characteristics. Statistical analyses were performed using SAS Enterprise Guide 9.2 (Cary, NC).

## Results

Overall, samples were from older patients who were nondrinkers, half of whom were female, and half of whom had smoking exposure. Twenty percent of samples were derived from diabetic patients.

Table 1 shows summary statistics for our tissue sample when divided into normal and PaC groups. In bivariate analyses, normal samples were significantly more likely to come from younger patients. There was a trend toward normal samples being derived from patients with less tobacco exposure.

**Table 1:** Summary Statistics for sample^a^

Characteristic	Normal Tissue ^b^	PaC ^c^	p ^d^
Age	59 (26-79)	64.5 (45-79)	0.007
Female	13 (62%)	28 (47%)	0.22
History of Tobacco Use	5 (28%)	27 (57%)	0.051
History of Alcohol Use	5 (28%)	20 (42.6%)	0.39
Diabetes Mellitus	3 (17%)	10 (21%)	1.00

^a^Table values are n (column %) except for age which is represented as median (range). Statistical significance was taken to be p≤ 0.05

^b ^N = 21 for age and female characteristics, n = 18 for tobacco/alcohol/diabetes due to missing data. Column percentages will not sum to 100%.

^c ^N = 60 for age and female characteristics, n = 47 for tobacco/alcohol/diabetes due to missing data.

Column percentages will not sum to 100%.

^d ^p-value calculated via Wilcoxon Rank-Sum (age), Chi square (female), or Fishers exact tests (Tobacco, Alcohol, Diabetes)

**Figure 2:**
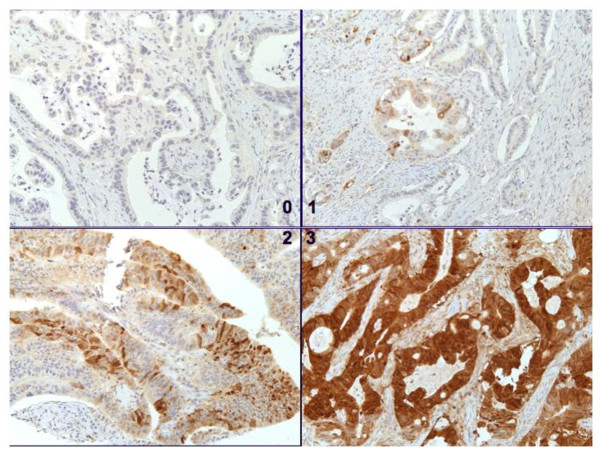
**FABP-1 Immunohistochemistry Staining Graded from 0-3**.

Figure 2 reveals representative pancreatic adenocarcinoma demonstrating no staining (upper left, 100x), 1+ staining (upper right 100x), 2+ staining (lower left, 100x), and 3+ staining (lower right, 200x) with FABP-1.

Table 2 describes our sample in terms of FABP-1 staining by normal tissue versus PaC. The majority of samples in normal and PaC groups did not stain for FABP-1. In bivariate analyses when FABP-1 staining was considered a binary variable, PaC samples were significantly more likely to stain for FABP-1. There was a trend towards statistically increased staining of PaC samples when FABP-1 staining was interpreted as a four-level score.

**Table 2:** Description of the Sample by Normal Tissue vs. Pancreatic Adenocarcinoma^a^

Characteristic	Normal Tissue ^b^	PaC ^c^	p ^d^
FABP-1 positive ^e^	1 (5%)	18 (30%)	0.02
FABP-1 Score			0.059
0: Negative	20 (95%)	42 (70%)	
1: 1-25%	0 (0%)	12 (20%)	
2: 25-75%	1 (5%)	5 (8%)	
3: > 75%	0 (0%)	1 (2%)	

^a^Table values are n (column %)

^b ^N = 21

^c ^N = 60

^d^p values represent Fisher's exact test

^e^FABP-1 positive defined as FABP-1 score ≥ 1.

In Table 3, FABP-1 staining was compared in normal and PaC groups, stratified for diabetes. Only 3 normal samples were derived from diabetic patients, and none stained for FABP-1. Because stratification of normals into diabetic/nondiabetic groups yielded no difference in the analysis described, the decision was made to present the normal samples as a single group of diabetics and non-diabetics.

**Table 3:** Description of the Sample by Normal Tissue vs. PaC with and without Diabetes^a^

Characteristic	Normal Tissue (n = 21)	PaC no DM (n = 37)	PaC-DM (n = 10)	p ^b^
FABP-1 positive^c^	1 (5%)	9 (24%)	5 (50%)	0.02
FABP-1 Score				0.02
0: Negative	20 (95%)	28 (76%)	5 (50%)	
1: 1-25%	0 (0%)	7 (19%)	3 (30%)	
2: 25-75%	1 (5%)	2 (5%)	1 (10%)	
3: > 75%	0 (0%)	0 (0%)	1 (10%)	

^a^Table values are n (column %). Due to missing data on diabetes status, the total number of patients in the PaC group was n = 47. PaC-DM represent samples from patients with pancreatic adenocarcinoma and diabetes. It was not possible to differentiate new-onset diabetics from chronic diabetics.

^b^p values represent Nonparametric Kruskal-Wallis ANOVA.

^c^FABP-1 positive defined as FABP-1 score ≥ 1.

Because the data failed to meet assumptions of normality and homogeneity of variances, we performed nonparametric Kruskal-Wallis ANOVA to compare FABP-1 staining in normal, PaC without DM (PaC no DM), and PaC with diabetes (PaC-DM) samples. PaC-DM samples represent tissue from patients with pancreatic adenocarcinoma-associated diabetes as well as PaC with coexisting chronic type II diabetes. The information to differentiate between these two groups was not available during chart review. Nonparametric analyses revealed a significant difference in FABP-1 staining (using both binary and FABP-1 score) between samples from normal, PaC no DM, and PaC-DM groups, with significantly increased staining as one moves from left to right in the Table 3. The PaC-DM group had the highest percentage of FABP-1 staining. Only one normal sample stained positively for FABP-1. There was no significant difference in age between samples from PaC no DM and PaC-DM groups that stained for FABP-1.

To elucidate relationships between the three groups, individual paired tests were conducted using the Wilcoxon Rank sum test with Bonferroni correction for multiple comparisons. There was significantly increased staining in the PaC-DM samples compared to normals (p = 0.004) and there was a trend towards significantly increased staining in the PaC no DM group versus normal (p = 0.07). There was no statistical difference in FABP-1 staining between PaC no DM and PaC-DM groups (0.09).

In logistic regression modeling, FABP-1 staining (binary) was significant associated with diagnosis of PaC with an odds ratio of 8.6 (95% CI 1.1-68, p = 0.004). Age was a confounder in this model. Patient gender did not modify the relationship between FABP-1 staining and diagnosis.

## Discussion

Using methods in integrative genomics, we predicted an association between FABP-1 and pancreatic adenocarcinoma and pancreatic adenocarcinoma with diabetes. Compared to normal controls, we found a significant positive association between increased FABP-1 staining and PaC on FFPE-TMA, strengthened by the presence of diabetes.

FABP-1 is a cytosolic chaperone protein that is expressed in the liver, kidney, lung, and GI tract [24]. The exact role of FABP-1 has yet to be elucidated. The association of FABP-1 with pancreatic adenocarcinoma and diabetes is of interest. Plausibility of FABP-1 as biomarker for cancer comes from previous literature suggesting an association with colon, gastric, and liver cancers. The literature is variable, but loss of FABP-1 expression in colonic tissue has correlated with progression from normal colonic tissue to adenoma to carcinoma [25]. Negative FABP-1 expression in colon cancer metastases has been associated with decreased survival, and low FABP-1 expression in colon cancer has been associated with lymph node metastasis [26,27]. FABP-1 gene expression has been identified as a marker of circulating colorectal tumor cells via RT-PCR [28]. On the contrary, one study associated low FABP-1 expression in colon cancer with increased survival [29]. FABP-1 is highly expressed in gastric intestinal metaplasia and a subset of gastric adenocarcinoma [30]. In one study, half of 62 hepatocellular carcinoma samples contained L-FABP immunoreactive tumor cells [31]. A single study of gene array analysis in a cohort of 21 patients revealed FABP-1 overexpression (16x) in PaC compared to chronic pancreatitis and normal pancreatic tissue, though no further testing was performed to validate this association [5]. Another group using bioinformatics methods also identified FABP-1 as a candidate molecule potentially associated with PaC, though again, no further validation was performed [32].

Plausibility of FABP-1 as a biomarker for diabetes comes from animal and human data. FABP-1 null mice demonstrate age dependent weight gain, though may be protected from obesity and hepatic steatosis when fed a saturated fat diet [33-36]. In humans, high levels of FABP-1 in placental homogenates have been associated with gestational DM [37]. Two genes with variants leading to Maturity Onset Diabetes of the Young (MODY), Hepatic nuclear factor (HNF)-4alpha and HNF-1alpha, are known to directly bind to the promoter region of FABP-1 [38]. Effects of FABP-1 are modulated through PPAR-gamma, the molecular target for the thiazolidinedione class of diabetic medications [39]. In humans, FABP-1 polymorphisms have been associated with increased levels of fasting triglycerides/LDL in females and urinary FABP-1 excretion is associated with hypertension and coronary artery disease risk factors [40,41]. With its up-regulation in various forms of cancer and effects on metabolism, it is plausible that FABP-1 could be a biomarker for pancreatic cancer and pancreatic cancer-associated diabetes.

To our knowledge, this is the first study to attempt to validate the FABP-1 association for PaC and PaC with DM. The intent of our study was to examine whether a computational approach could identify proteins associated with a particular disease, as a preliminary step in biomarker discovery. As mentioned, we narrowed our search to proteins present in serum/urine because of the postulated presence of a circulating diabetogenic factor that exerts peripheral effects. Also, a diagnostic test would necessarily be a serum/urine assay.

Of note, FABP-1 has been in detected in animal and human serum/urine and commercial ELISA-kits are available for FABP-1 serum/urine detection [42-47]. Possible mechanisms for FABP-1 presence in the serum could involve cell lysis or cellular secretion. The discovery of a biomarker for PaC-associated DM would be of immense value for the early detection and treatment of PaC. Association by immunohistochemistry alone, however, does not necessarily translate to diagnostic utility nor causality. We have no evidence to prove that FABP-1 is responsible for the pathogenesis of diabetes. Future experiments will involve larger sets of both pathologic and serum samples, with closely phenotyped patient samples to further explore this association.

Global gene expression analysis in patients with PaC-associated DM is a complimentary and contributory approach to biomarker discovery using integrative bioinformatics. Gene expression analysis could confirm the FABP-1 overexpression we predict. There is no published literature that specifically investigates the gene expression of tumor tissue in PaC-associated DM. Future work will be important to fill that knowledge gap.

There are several limitations of our study. When considered a binary variable, FABP-1 staining was defined as a score ≥ 1. A more stringent cutoff for FABP-1 staining would change our reported associated with pancreatic cancer. However, we do feel that in this pilot study, grouping pancreatic cancer groups by the presence or absence of FABP-1 staining was reasonable, given that only one normal sample stained positive for FABP-1 and that the degree of FABP-1 expression is likely to vary between tumors. The extent of FABP-1 staining will be explored in future studies with more patients samples that are better phenotyped. In multivariate analyses, age was noted to be a confounder of FABP-1 staining, though it should again be noted that only one sample in the normal group stained with FABP-1 and this sample was from a patient that was younger than the average age of the staining population in the PaC group. Rather than a true confounder, the association between age and FABP-1 staining may be a result of selection bias from our convenience sample or a spurious association. Larger studies are needed to further explore this relationship.

There are limitations of the bioinformatics technique itself, including potential low specificity for a given biomarker, the assumption that differential gene expression is necessarily reflected in plasma or urine, and that we excluded proteins currently not known to be detectable in serum or urine. Biomarker discovery using this methodology is necessarily limited by quantity, quality, and availability of public microarray datasets for a disease. However, it should be noted that these limitations have not precluded successful discovery of biomarkers for other diseases [12].

Our data collection was retrospective and subject to idiosyncrasies in chart documentation. We could not differentiate pancreatic cancer-associated diabetes from pancreatic cancer coexisting with chronic diabetes. The PaC-DM group presented represents true PaC-associated DM cases as well as those in whom the DM was unrelated to PaC, in unknown proportions. This distinction can be elucidated in future serum FABP-1 studies with more detailed available clinical information.

Our sample was a nonrandom small convenience sample, which likely contributed to the lower than published prevalence of diabetes in PaC, the age difference between normal and cancer groups, and the fact that we were not adequately powered to run a matched analysis [3]. However, one would expect nondifferential misclassification and a small sample size to skew results towards the null. The fact that a significant association between FABP-1 staining PaC and PaC with diabetes was found suggests that the true association is stronger. It would have been informative have more normal samples from diabetics to confirm lack of FABP-1 staining.

Finally, our normal samples were representative of histologically normal tissue that often did come from organs that had pancreatic pathology. We cannot rule out concerns of tumor microenvironment, though it should be noted that histologic normalcy is the basis for adequate surgical margins during tumor resection, and that this technique is standard practice in construction of microarrays and has yielded reproducible results for other types of cancer/normal tissue [22,23]. Future studies will use a control group of patients without pancreatic disease. Only one pathologist scored immunohistochemistry; however, he has several years experience. Our study would have been strengthened by having at least two independent pathologists read and score FABP-1 staining.

## Conclusions

In conclusion, our study was proof of concept that a translational bioinformatics approach has potential to identify novel disease associations in gastroenterology. Further experimental and clinical research is required to understand the true relationship between FABP-1, pancreatic cancer, and PaC-associated DM.

## Competing interests

The authors declare that they have no competing interests.

## Authors' contributions

RNS, AJB, and PJP conceived of the study and its design and participated in drafting the manuscript. AJB and JTD performed the bioinformatic work. KDM performed the tissue microarray work. RP scored the stained tissue microarrays. All authors read and approved the final manuscript.

## Pre-publication history

The pre-publication history for this paper can be accessed here:

http://www.biomedcentral.com/1471-230X/11/5/prepub
